# An assessment of the quality of care provided at primary health care centres in camps for internally displaced persons in Iraq in 2018

**DOI:** 10.1186/s13031-021-00402-4

**Published:** 2021-09-08

**Authors:** Muhammad Fawad Khan, Daniel Jeannetot, Kamal Sunil Olleri, Mirjam Bakker, Altaf Sadrudin Musani, Adham Rashad Ismail Abdel Moneim, Wael Hatahit, Prisca Zwanikken

**Affiliations:** 1 WHO Country Office – Iraq, Baghdad, Iraq; 2 Erasmus Medical Centre, Rotterdam, The Netherlands; 3 KIT Royal Tropical Institute, Amsterdam, The Netherlands

**Keywords:** Internally displaced persons, Quality of care, Camp, Iraq, Primary health care, Complex emergencies

## Abstract

**Introduction:**

The humanitarian crisis in Iraq remains one of the largest and most unstable in the world. In 2014, over 2.5 million civilians were displaced in Iraq; between 2015 and 2017 more than 3 million people continued to be displaced. While health-related research concerning internally displaced persons (IDPs) population has been conducted in many settings, very few have looked at the quality of care delivered in primary health care centres (PHCC) inside camps. The objective of this operational research is to assess the quality of health care services at PHCC in operational IDP camps supported by local and international NGOs (humanitarian partners) as well as the Directorate of Health (DoH) in Iraq at baseline and after 6 months.

**Method:**

A framework based on five components was used to assess quality of care by assigning a quality-of-care index score. Using a longitudinal design; data were collected through observations of facilities and of patient consultations, as well as health worker and patient exit interviews, in static PHCC in operational IDP camps of Iraq during two different phases: in June (n = 55), and December 2018 (n = 47). These facilities supported more than 500,000 IDPs. Descriptive and statistical analyses were conducted, and the results compared.

**Result:**

For all camps (n = 47), the average overall quality of care index score increased between the two phases. No specific type of organisation consistently provided a better quality of care. The camp size was unrelated to the quality of care provided at the respective facility. The domain indicators “Client Care” and “Environment and Safety” mostly related to the variation in the general assessment of quality. Patient satisfaction was unrelated to any other domain score. Compared at 0 and after 6-months, the quality of care index score between the type of organisation and governorate showed that feedback positively impacted service delivery after the first assessment. Positive differences in scores also appeared, with notable improvements in Client care and Technical competence.

**Conclusion:**

Humanitarian partners and the DoH are able to provide quality care, independent of camp size or the number of camps managed, and their cooperation can lead to quick improvements. This research also shows that quality of care assessment in emergency settings can be carried out in formal IDP camps using non-emergency standards.

**Supplementary Information:**

The online version contains supplementary material available at 10.1186/s13031-021-00402-4.

## Introduction

After the Emergency Relief Coordinator of the Inter-Agency Standing Committee (IASC) declared Iraq to be a “Level 3 emergency” in 2015, the highest on the humanitarian crisis scale, emergency funding channelled through Humanitarian Response Plans was made available to provide assistance and relief to the Iraqi population through the Cluster Approach.^1^ Over a period of 5 years, more than six million people were displaced [1], 18% of the population. As early as 2016, the situation had partially stabilised and people started returning home [1]. In December 2017, the government of Iraq officially declared victory over the Islamic State in Iraq and Levant (ISIL) group [2]. In 2019, more than 1.7 million people were still displaced and in need of assistance with shelter, protection, and health services among other basic needs, with almost 30% still staying in camps [3].

Jordan et al. point at the large gap in measuring quality both at the point of care and at the health system level [4]. In humanitarian contexts globally, many aspects of health have been assessed in the literature for refugee or internally displaced persons (IDPs), camps setting (e.g., health status, access, acceptability) [5–9], however quality of care has not been directly assessed.

### Literature review

Quality of care is widely researched in high-income countries, but studies have rarely been conducted in emergency or post-emergency settings [10]. The focus of most research on health care provision in emergency and post-emergency settings is on outcomes indicators [11–16] where the priority is mortality reduction. Nonetheless, the concept of quality of care exists in key documents: the SPHERE standards [17], which provides minimum standards for all aspects in humanitarian response, e.g. health service delivery, are used to benchmark the quality of care provided. Regarding health service delivery, SPHERE stipulates that “People have access to integrated quality healthcare that is safe, effective, and patient-centred,” but does not mention the quality of care as understood in non-emergency contexts [17] and as defined by the WHO: “the extent to which health care services provided to individuals and patient populations improve desired health outcomes. In order to achieve this, health care must be safe, effective, timely, efficient, equitable and people-centered” [18]. In its 2014–2018 Global Strategy for Public Health, UNHCR set its first objective to be “[To] Improve access to quality primary health care programmes” [19]. In fact, the SPHERE standards have not all been based on evidence, but mostly on what is considered best practice by the humanitarian community, since evidence can be difficult to generate, verify, and generalise in such settings [20, 21]. Beyond the quality of care, the focus has more often been on the *quality of life* in camps, either for refugees or IDPs, which includes aspects of health care services [22–25]. Most studies in IDP camps measure quality as set by SPHERE standards, others focus on the outcomes of specialised care [26–29]. Only a few studies attempt to consistently evaluate the quality of health care provision using SPHERE standards. They include other components such as WASH or nutrition, extending beyond the scope of health service delivery quality [11, 30, 31]. Interestingly, instead of using SPHERE standards, Kersten et al. used quality as conceptualised by Donabedian, to demonstrate the feasibility of assessing the quality of care in emergency settings [10]. The necessity to move beyond SPHERE standards to assess the quality of care in emergency settings is also based on ethical principles: patients, including in humanitarian crises, have a right to quality of care.

The scarcity of evidence on the quality of care in health facilities in IDP camps joins the ongoing discussion regarding the lack of (quality) evidence from interventions in humanitarian settings. More broadly, the call for better evidence to feed practice and standards in (post-)emergency settings has been heard for many years, but slow to materialize in practice [32–36].

Quality of care has been defined in different ways in the literature [34–40]. For the purpose of the rapid assessment, the concept of quality of care used was defined by the Primary Health Care Initiative (PHCI) Quality Improvement Handbook of the Hashemite Kingdom of Jordan, because, in addition to providing a conceptual definition, it also provides a tool to operationalise the definition. This study therefore uses the same concept. The definition is: “the proper performance (according to standards) of interventions that are known to be safe, that are affordable, and acceptable to the society in question, and that have the ability to improve health outcomes and meet or exceed client expectations” [41]. The situation called for a rapidly implementable, context-sensitive, and deployable tool which was already operationalized.

There is an evidence gap in the quality of care in primary health care facilities in IDP camp settings [4], which this research attempts to fill. In 2018, the Health Cluster, led by WHO, initiated assessments of the quality of primary health care services provided at primary health care centres (PHCCs) in IDP camps in Iraq. The main purpose of these assessments was to give feedback to improve the quality of care provided by implementing partners, the secondary purpose, was to assess the feasibility of measuring the quality of care in IDP camps through non-emergency standards. A summary of the results is available in the public domain [42, 43].

### Objective

The primary objective of this operational research was to assess the quality of health care services provided at primary health care centres (PHCCs) in existing IDP camps in Iraq in 2018 at two points in time and provide recommendations on how to study quality of care in IDP camps to support future implementation. Secondary objectives included assessing how governorate, implementing partners, and camp population size could impact the quality of care of PHCC in IDP camps in emergency situations.

### IDP camps organisation in Iraq

Health services in Iraqi IDP Camps were part of the camp management tool kit developed by the global Camp Coordination and Camp Management (CCCM) Cluster, which support standards and policies in managing displaced population in camps [44]. Health services in formal camps were run by local or international NGOs, some by the DoH (Governorate’s Directorate of Health), all of which are part of the Health Cluster. All camps had one primary health care centre on-site, with staff provided by the Iraqi Ministry of Health and managed on-site by implementing partners. Camps with more than 10,000 individuals had increased staff capacity along with satellite health posts inside the camps and ambulatory maternal and childcare services. While PHCCs provide basic health care services, patients in need of reproductive and maternal health were directed to specific services for reproductive and maternal service organised separately. On average, staffing at PHCCs consisted of four general physicians, two nurses per general physician, a lead pharmacist, and a lead laboratory technician. In the most populous camp, a maximum of eight general physicians were present. The PHCCs only provided basic services, therefore when patients required more care, they were referred by the health partner to the nearest secondary–level health facility outside the camp by bus or ambulance and brought back after treatment.

## Methodology

### Design

This study had a longitudinal design using PHCC; data collection took place in June (Phase 1) and December (Phase 2) 2018. The findings of Phase 1 were provided to implementing partners to improve quality of care before Phase 2 took place.

### Indicators and data collection tool

The definition of quality of care in the conceptual framework provided domains to deconstruct quality of care as shown in Table 1 and allowed it to be measured.

**Table 1:** Conceptual framework quality of care.

Technical competence	Staff are competent to provide services for general and reproductive health conditions
Client care	The center provides information about services, health, and follow up care to ensure understanding compliance, confidentiality, and satisfaction
Management	The center plans, staffs, organizes and implements health delivery services to ensure efficiency and effectiveness for clients, community, and staff members
Environment and safety	The center provides a client friendly, accessible and safe environment
Satisfaction	The center meets staff and client expectations and needs by providing well planned, appropriate, safe, and effective services

*Source*: Quality improvement handbook for primary health care, 2004

The conceptual framework is operationalised by a list of tested and tailored tools [41, 45]. The questions were adapted for reasons of efficiency and available resources in the given context. The data collection tool consisted of observations and interviews. Observations were a facility checklist with 13 items, and clinical care checklists for all health worker types during interactions with patients (Additional file 1). The interviews consisted of two subsets: one for all health workers with the same 5 questions, and one for patients with 22 questions. Questions in both the observation and interview sets were closed, in the form of Yes/No, or a three level Likert scale type (Additional file 1). Data collection took between 30 and 60 min per observation/interview.

### Unit of analysis, population and sample

The unit of analysis were the IDP camp. Each camp was served by one general primary health facility, included in this study. The sample population was composed of all the camps listed in the CCCM Camp master list for Iraq. The sample included 55 camps in June 2018, and 47 in December 2018. The number of camps fell because of a decreased IDP count and the closure or consolidation of camps. Between June and December, 46 camps were the same and therefor comparable between the datasets, while the remaining camps were only existing in that specific period. General PHCCs were selected for the survey. Reproductive care facilities, which were separate facilities in the IDP camps in Iraq, although some reproductive health services were available in the PHCC, were excluded.

The samples of services providers and patients per facility consisted of four service providers: the on-duty physician and nurse in examination room one, the lead pharmacist, and the lead laboratory technician. Six patients were interviewed for the post-consultation questionnaire. The first patient leaving a consultation on the day of data collection was selected as the first respondent. The five others were picked at random using the roll of a die, selecting the nth patient at the exit of the facility according to the number rolled. Fourteen patients across the 55 camps in Phase 1 did not participate, all solicited patients participated in Phase 2. If a patient refused to participate, the next patient to come out of consultation was selected.

### Data collection protocol

Data were collected in all PHCC run by the Health Cluster partners. The assessment took 4–6 days per governorate. The complete assessment took 1 day per facility. The data were collected using the Kobo Tool Box [46] a smartphone/tablet data collection tool. The observation checklists were in English, and the interviews were conducted in Arabic or Kurdish. The answers were collected in English. All enumerators knew English/Arabic/Kurdish and could translate the results accordingly.

The data collection was performed by teams comprising two trained enumerators from the Iraqi Red Crescent Society (IRCS), including a medical doctor for the observation assessment, and a pharmacist or dentist for the interviews. The teams were composed of one male and one female. There was a specific protocol for gender-disaggregated data collection. IRCS was selected as they did not support any PHCC services in the camps, limiting the chances of bias. Population and camp data were obtained from the IOM data matrix [47] as were base characteristics of the operation. Average family size, number of families per camp and female to male ratio were estimates. Age and sex-disaggregated data were not available because of setting constraints.

Patients seeking specific maternal and reproductive care were not captured in the patient exit interviews because the focus was on general primary care services. Thirteen INGO-supported camps were either closed, consolidated, or transferred to NGOs between Phase 1 and 2.

### Data management and analyses

The data were cleaned and verified using Microsoft Excel, and analyzed using R and Rstudio [48, 49]. The data went through a visual and descriptive inspection, before being analyzed through linear regressions. No missing data point was found.

Each domain of the framework was assessed by a different set of questions, rated independently in percentages. The five domains were combined into an Index score from 0 to 100%, with 100% corresponding to the highest standard of quality of care.

The domain scores were calculated based on the average answers per questions provided by a set of domain-specific questions and observations. The questions and observations were coded so that a positive answer would increase the score of the domain (“Yes” = 2, “No” = 0 and “None” = 0, “Partial” = 1, “All” = 2). Within the domains, the questions were equally weighted. The maximum per domain was 100% meaning that all standards of care measured by the questions were met. The Index score was composed of each domain score average, equally weighted.

The use of the Index score and of the independent domain scores provided an analytical framework which allowed the comparison of different units of analysis: camps, governorates, and type of supporting organisation (NGO, INGO, Department of Health) using averages per unit of analysis.

Population and base camp data were reported, then Index and domain scores were provided by Governorate and type of organisation. Phase 1 (June 2018) results were analysed (n = 55) and the different scores for the Governorate and type of supporting organisation were assessed. Then Phase 2 (n = 47, December 2018) was compared to Phase 1, with a focus on the same camps between phases (n = 46). The third step considered all camps between the two phases (n = 102), specifically looking at the domain contribution to the Index Score through linear regressions, R^2^ and *p* values were reported. Furthermore, the relationship between camp size (IDP count) and index scores was evaluated. Statistical analyses (T-test) were conducted to compare means between phases and assess the significance when appropriate.

## Results

### Population data, camp characteristics

In Phase 1, there were 535,253 individuals spread over 55 camps, distributed in 89,235 families with an average of 6 members per family. The 55 camps were spread over 20 districts located in 8 governorates. On average, 51% of the individuals were females. The governorate of Ninewa contained most camps (n = 19), followed by Dahuk (n = 16). Together these two governorates accounted for almost 80% of the IDP population located in camps. Ninewa alone accounted for 52%, with the biggest camp having more than 44,000 individuals (Qayyarah Airstrip) in the governorate. The smallest camp was located in the governorate of Sulayamniyah, (174 individuals). The median camp population was 5910 individuals. Out of the 55 camps, 24 PHCCs were run by NGOs, 22 by international NGOs (INGO), and 9 by the Directorate of Health (DOH). INGOs supported 169,500 IDPs, i.e., 31% of the total IDP population in camps, NGOs supported 341,391 IDPs. In Phase 2, 47 camps were operational, the total number of individuals was 513,978 (85,633 families), 51% females, with median camp size of 8826 individuals. The governorate of Ninewa and Qayyarah Airstrip were still the most populous.

### Governorate and type of supporting organisation results

A summary table (Table 2) describes all domains and Index score by governorate, by type of supporting organisation, and phases.

**Table 2:** Summary table of average index score and domains by (**a**) governorate and (**b**) type of supporting organization

	IDP COUNT		# CAMPS		Index score (%)		Environment and safety (%)	
	Phase 1	Phase 2	Phase 1	Phase 2	Phase 1	Phase 2	Phase 1	Phase 2
*(a) Governorate*								
Anbar	38,465	27,546	6	3	53.1	80.2	53.7	79.6
Dahuk	151,896	152,136	11	11	57.4	58.3	66.7	62.1
Diyala	7332	4068	3	1	64.8	43.1	87.0	77.8
Erbil	19,062	10,488	4	1	50.6	72.4	47.2	66.7
Kirkuk	17,898	13,278	5	4	57.1	62.3	64.4	80.6
Ninewa	275,802	278,322	19	19	54.7	69.8	61.1	75.7
Salah al-Din	4782	7506	2	3	57.5	56.2	72.2	79.6
Sulaymaniyah	20,016	20,634	5	5	51.8	64.7	60.0	73.3
Average					55.9	65.2	64.1	74.4
Total	535,253	513,978	55	47				
*(b) Type of supporting organisation*								
DOH	24,360	41,430	9	7	61.7	63.7	71.6	79.4
INGO	169,500	75,180	22	9	52.1	74.3	54.0	88.9
NGO	341,393	397,368	24	31	55.9	62.9	66.7	67.0
Average					55.9	65.2	64.1	74.4
Total	535,253	513,978	55	47				

	Client care (%)		Technical competence (%)		Management (%)		Satisfaction (%)	
	Phase 1	Phase 2	Phase 1	Phase 2	Phase 1	Phase 2	Phase 1	Phase 2
*(a) Governorate*								
Anbar	48.6	79.0	51.5	65.2	47.9	83.6	63.8	93.8
Dahuk	59.6	47.4	50.4	50.4	53.1	64.6	57.0	66.8
Diyala	59.5	21.4	50.0	9.1	55.2	49.1	72.2	58.3
Erbil	53.9	75.0	54.5	86.4	38.2	70.4	59.1	63.7
Kirkuk	60.5	62.8	44.5	59.1	52.3	41.4	63.7	67.7
Ninewa	53.8	71.3	49.5	72.5	50.5	61.4	58.5	68.1
Salah al-Din	51.8	55.6	61.4	62.1	53.2	38.0	49.1	45.8
Sulaymaniyah	47.6	66.0	42.7	57.3	51.9	61.3	56.7	65.6
Average	54.4	59.8	50.6	57.7	50.3	58.7	60.0	66.2
*(b) Type of supporting organisation*								
DOH	61.9	62.2	52.0	54.5	55.9	51.2	67.3	71.3
INGO	51.0	79.6	50.8	80.3	48.2	60.2	56.6	62.4
NGO	55.4	58.2	47.7	58.9	50.6	62.4	59.3	68.0
Average	54.4	59.8	50.6	57.7	50.3	58.7	60.0	66.2

#### Phase 1

In Phase 1 at camp level, the average Index score was 55.9%, with the lowest camp scoring 36.7% and the highest 77.2%. The lowest score was recorded in the governorate of Sulaymaniyah, where the facility was supported by an INGO, 15 percentage points lower than the governorate average. The highest scoring camp was found in the governorate of Dahuk, supported by an INGO.

At the governorate level, the Governorate of Diyala scored the highest on the overall average performance (Index Score = 65%) and Erbil the lowest with 50.6%. Diyala hosted three camps, and all the PHCC were supported by the DoH. In Erbil, there were four camps, all these PHCC in the camps received INGO support.

According to Phase 1 composite scores, health facilities supported by INGOs provided the lowest overall quality of care with an average of 52.1% on the Index score, and scored the lowest on all domains, except Technical Competence when compared to the Directorate of Health or NGOs. However, the differences between INGOs and other providers was not significant (*p* > 0.1).

**Fig. 1:**
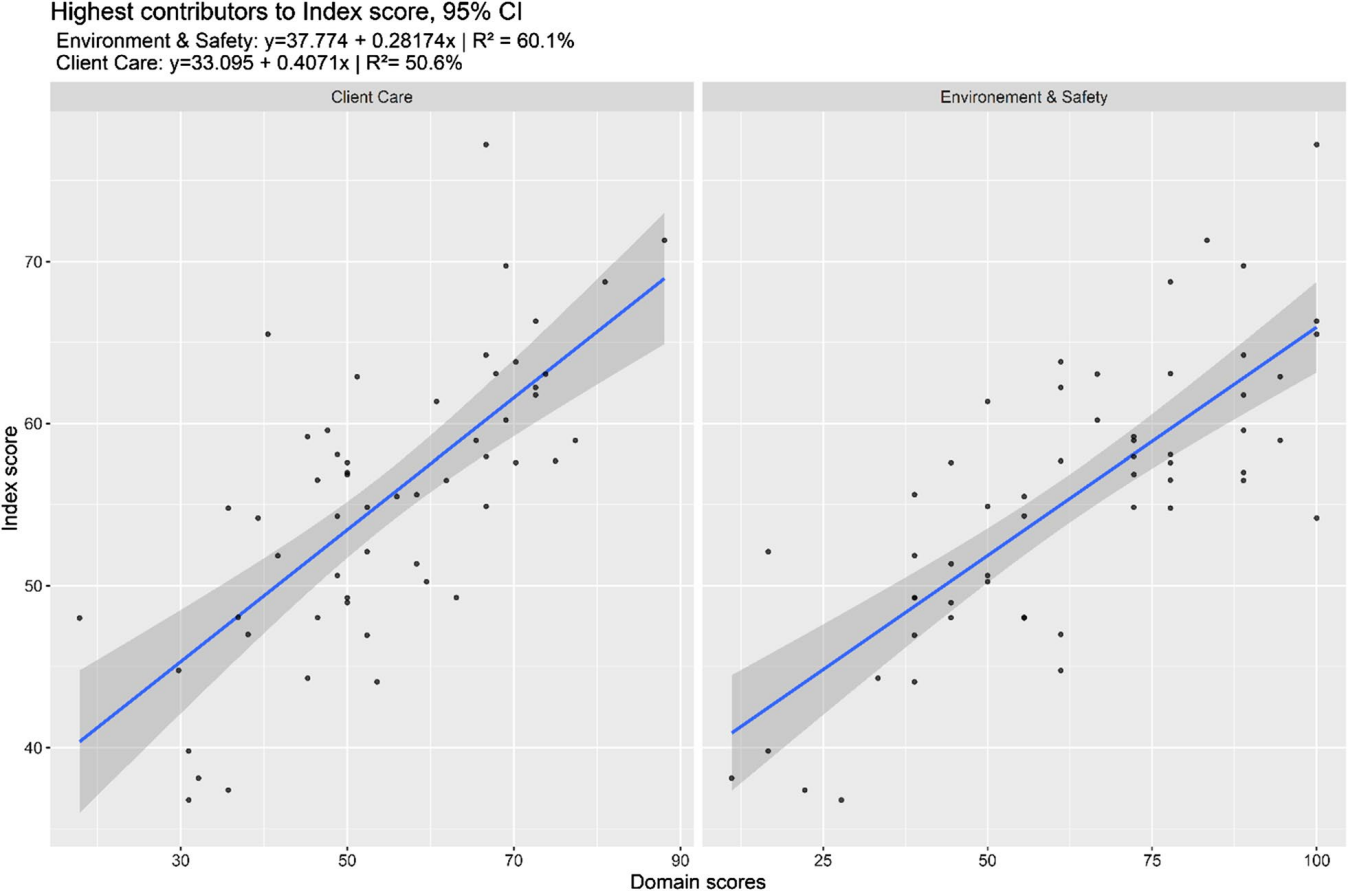
Highest contributors to index score phase 1

In Phase 1, the Client Care (CC) and Environment and Safety (ES) domains contributed most to the Index Score, as shown in Fig. 1. The ES domain accounted for 60.1% and Client Care for 50.6% of the variation in the overall Index score (*p* < 0.001). Both related mostly to observational aspects as they were only measured by facility and consultation observations. Management explains about 22.7%, Technical Competence 19.3% (both significant at *p* < 0.01), but Satisfaction was not significant and only explained 6.8% of the variance.

#### Comparison phase 1–phase 2

In Phase 2, the average Index score was 65.2%, with a maximum of 89.8% and a minimum of 33.6%. For all camps (n = 102), the overall average index score had increased between the two phases by 10 points. The highest-scoring camp was located in the governorate of Ninewa, supported by an NGO. The lowest scoring camp was in the governorate of Dahuk, which was also supported by an NGO. The camp scored 0% on Technical competence and 6% on Client care. Compared to Phase 1, the highest scoring camp scored 12 percentage points higher, with more than 8s000 IDPs. The profile of the lowest-scoring camp in phase 2 was very different of that from Phase 1: the number of IDPs was 24 times bigger than the lowest-scoring in Phase 1. The lowest-scoring camp of phase 1 had been consolidated into another camp. The highest-scoring in Phase 1 scored 70.7% in Phase 2, a decline of 10%. All implementing partners increased their index score between the two phases, with the highest increase for INGO from 52 to 74%, changing it from the lowest-scoring in Phase 1 to the highest-scoring in Phase 2.

**Fig. 2:**
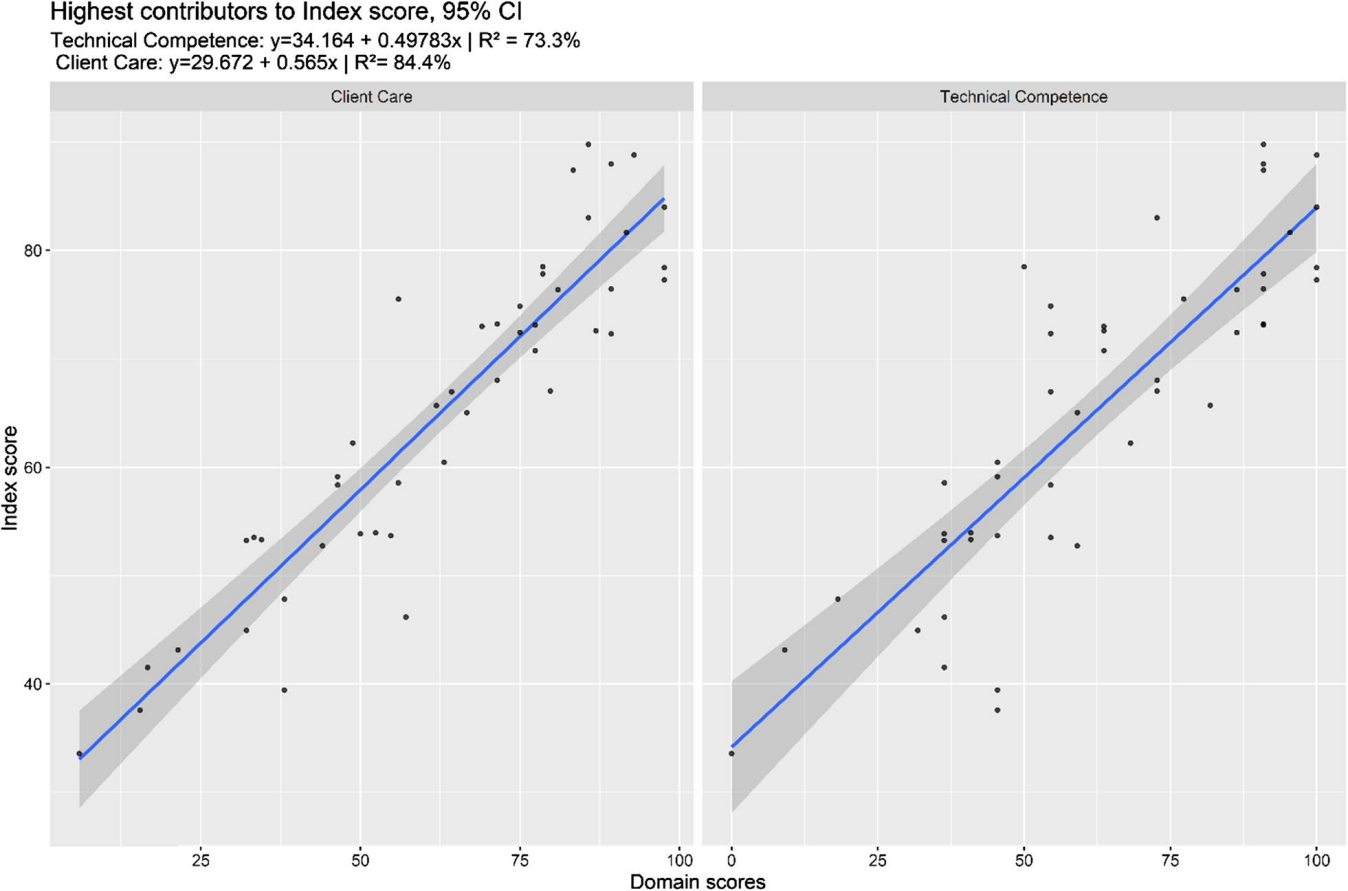
Highest contributors to index score phase 2

Alongside, the contributions of the different domains to the Index Score also changed compared to Phase 1. As shown in Fig. 2, Technical Competence (R^2^ = 60.1%) and Client Care (R^2^ = 50.6%) explained most of the variation in the Index Score (*p* < 0.001), and from a visual inspection, the dispersion was also lower than in Phase 1. The fact that the dispersion was lower could indicate that overall quality of care was being standardized and, therefore, that, on average, the camps were reaching the same standards of care as measured by the different domains. The analysis also showed that the satisfaction of patients increased and remained high from phase 1 to phase 2.

#### Differences between camps in phase 1 and phase 2

Out of the 55 camps in Phase 1, 46 remained open and functional in Phase 2, and there was one new camp (Salah al-Din Tikrit, Al-Alam Camp 1) in Phase 2. Table 2a and b show variations in the Index Score and the domains. The Governorate of Anbar showed the highest increase in scores with an average 30.6 percentage point increase for the three camps that remained open. On the other hand, Diyala, which lost two of its three camps from Phase 1, the Index score decreased by an average of 20 percentage points. The highest increase in domain score was in Satisfaction with a 41.7 percentage point increase in the Governorate of Erbil. Of the governorates that did not see any change in camp numbers (Dahuk, Ninewa, Sulaymaniyah), all scores increased.

In terms of the type of organisation in Phase 2, a large number of INGOs were not handling camps anymore (13 camps fewer) while NGOs took on 7 more camps. In general, the different domains, as well as the index score, increased by more percentage points for INGOs than for NGOs. In concordance to both Phase 1 and Phase 2 for all camps, the main contributing domain to the index score and the index score difference remained ‘Client Care’ (r^2^ = 81%), followed by ‘Environment & Safety’ and ‘Technical Competence’ (r^2^ = 65% and 64.1%) all significant at *p* > 0.001. The differences between Phase 1 and Phase 2 are shown in Table 3.

**Table 3:** Point difference between phase 1 and phase 2

Governorate	Point difference between phase 1 and phase 2 (phase 2–phase 1)						
	# Camps difference	Index score	Environment and safety	Client Care	Technical competence	Management	Satisfaction
Anbar	− 3	27.1	25.9	30.4	13.7	35.7	30
Dahuk	0	0.9	− 4.6	− 12.2	0	11.5	9.8
Diyala	− 2	− 21.7	− 9.2	− 38.1	− 40.9	− 6.1	− 13.9
Erbil	− 3	21.8	19.5	21.1	31.9	32.2	4.6
Kirkuk	− 1	5.2	16.2	2.3	14.6	− 10.9	4
Ninewa	0	15.1	14.6	17.5	23	10.9	9.6
Salah al-Din	1	− 1.3	7.4	3.8	0.7	− 15.2	− 3.3
Sulaymaniyah	0	12.9	13.3	18.4	14.6	9.4	8.9
Average		7.5	10.4	5.4	7.2	8.4	6.21
*Type of supporting organisation*							
DOH	− 2	2.0	7.8	0.3	2.5	− 4.7	4
INGO	− 13	22.2	34.9	28.6	29.5	12	5.8
NGO	7	7.0	0.3	2.8	11.2	11.8	8.7
Average		10.4	14.3	10.6	14.4	23.5	6.4

### Contribution to index score, camp size, and distribution of scores

**Fig. 3:**
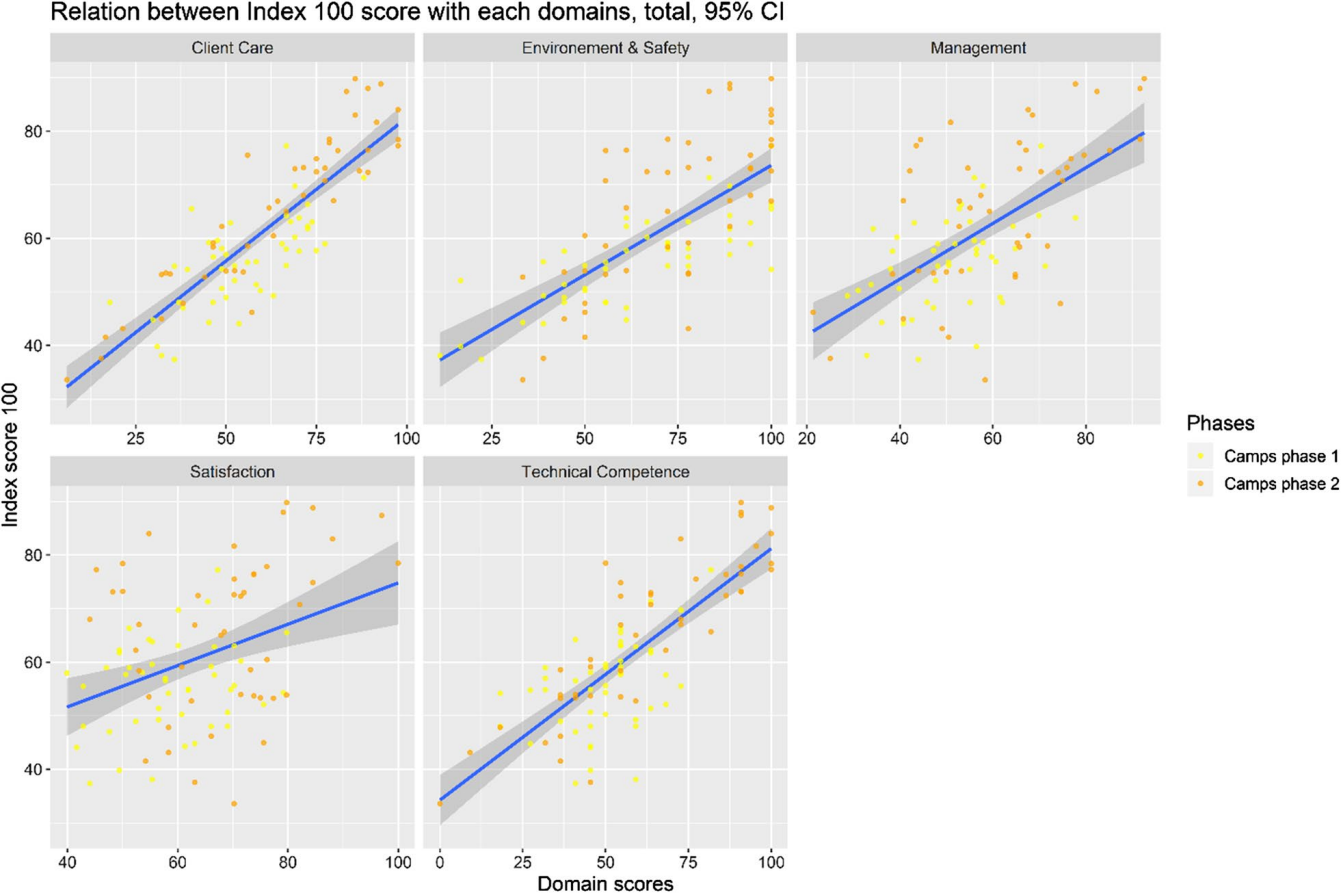
Domains contribution to index score, both phases

In general, when looking at all camps in both Phase 1 and Phase 2 (n = 102) and comparing which domain contributed most to the total score, ‘Client Care’ contributed most and with very little dispersion. On the other hand, ‘Management and Satisfaction’ seemed to be the least informative for the Index score (Fig. 3).

Patient satisfaction and camp size were also positively correlated but not significant (r^2^ = 2.4%, *p* > 0.1). A potential reason to explain such a pattern is that as the camp grows, more resources are mobilized. At the time of data collection, the camps and health facilities had already been running for some years, therefore, it is possible that issues were previously resolved. The same relation was observed between camp size and the other domain scores, yet the ‘Satisfaction’ score seemed unrelated to all other domains.

**Fig. 4:**
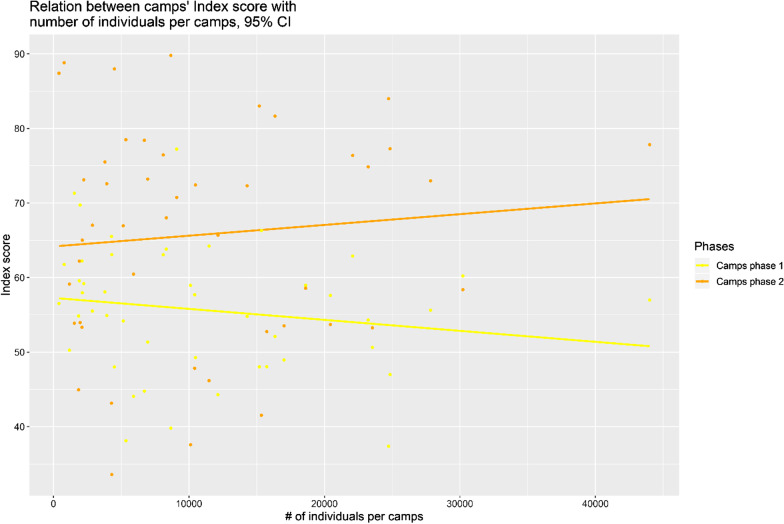
Relation between quality of care (index score) and number of individuals, both phase

A critical question regarding camps, in general, was whether the number of people per camp impacted the quality of care delivered, as the number of care providers and facilities were only increased by a small amount. As shown in Fig. 4, there was a very slight slope between the overall quality of care and the number of individuals in camps. However, if when removing the two outlying points (not shown; Qayyarah Airstrip Camp Phase 1 and 2), the relation became flat. We expected no further relation between the number of individuals and the quality of care provided.

## Discussion

This study was one of the first to evaluate the quality of care provided to IDPs in camps in emergency settings and we observed that the provision of quality care is correlated to the patient’s experience as well as environmental and safety factors. We also observed that the size of the camp or the type of organisation were not related to the measurement of quality of care. Below is discussed how governorate and type of organisation specificities may explain variances in results. The second part enquires about the type of indicators used here, as well as their interactions. Finally, the situation of quality of care in the Iraqi context and humanitarian settings are touched upon.

### Governorate and type of organisation performance

The differences in scores between governorates could be partially explained by the distribution of IDPs, the oversight by the respective DoH, and the number and capacity of partners (NGOs, INGOs). On average, the governorates of Ninewa and Dahuk were performing well in regard to the number of IDPs they have. It was worth noting that governorates with the lowest number of IDPs were the only two governorates for which the Index score dropped between Phase 1 and Phase 2. This could be attributed to access and security related issues, local capacity, as well asthe willingness of the DoH to oversee the quality of care in these camps.

INGOs, the lowest-scoring type of organisation in Phase 1, became the highest-scoring in Phase 2, apparently being the best able at incorporating the findings from Phase 1 into Phase 2 to improve the quality of care. According to respondents, local NGOs addressed most patients needs in both phases. However, while they outperformed other types of organisations in Phase 1, they became the lowest-performing in Phase 2.

### Scores, index, and their interactions

Out of both phases and for all camps, ‘Client care’ and ‘Environment and Safety’ appeared to be the most important domains contributing to the general quality-of-care score. Interestingly, direct satisfaction measurement showed little contribution and no significance to the Index Score. Some domains did not include questions measuring the same issues between interviews and observations due to design constraints. ‘Environment and Safety’ measure basic amenities (e.g., provision of water or clean toilets) for clients. Similarly, ‘Technical Competence’ was also measured mostly through observation. Finally, the domain contributing most, ‘Client Care’, collected observation pertaining to the patients’ experiences (see Additional file 1). It could imply that the experience of the patient in terms of quality in the facility and the feeling of being properly taken care of by the provider are crucial in providing quality of care. The literature in this regard, seemed to mostly corroborate this finding [27, 50–52], however, it cannot be fully answered here.

The lower dispersion of Client Care and Technical competencies scores seen in Fig. 2 could indicate that overall quality of care was being standardized and, therefore, that, on average, the camps were reaching the same standards of care as measured by the different domains. The analysis also showed that the satisfaction of patients increased and remained high from Phase 1 to Phase 2. The construction of the ‘Satisfaction’ domain was conceptually difficult, and the selection of questions composing the domain may explain why it did not relate to any score. Conceptual and theoretical issues relating to the scores were partially explained by the fact it was an operational research with the primary purpose to inform and improve quality-of-care delivery in PHCC. Additionally, the specific context may have led IDP not to be critical of the services received, skewing the results. In fact, the war in Iraq in 2003 and its long-term consequences severely weakened the health system [27, 50–56]. More broadly, a current debate in emergency setting camps is that people inside camps have access to better services than people outside camps [3, 11, 25, 56, 57]. That can largely influence the perceived quality of care, and therefore the satisfaction of patients.

Quality of care remains an unexplored domain in emergency settings [4], where the SPHERE standards are widely used and only provide basic minimum criteria [20, 34, 58]. As many crises are lasting longer, the importance of assessing the quality of care becomes essential and this research shows that it is possible to measure the quality of care to IDPs beyond emergency standards. Insights from this short assessment have already been used by the Health Cluster to inform the health partners operating in camps and improve quality in PHCCs.

### Limitations

As a rapid general assessment of the quality of care, some questions and observations that could bring extra critical information have been left out to reduce the data collection time. First, the conceptual and operational definitions of quality of care were chosen for pragmatic and contextual reasons relating to the emergency of situation, the need for a rapid assessment, and to the cultural and economic proximity between the Jordanian and Iraqi contexts. Other definitions could have provided different insights. Second, while patients declared in general to be satisfied with the services, no questions about the waiting time of patients, often a critical factor in quality [59–61], was asked. More broadly, ‘people centeredness’, part of the WHO QoC definition, could not be adequately assessed here. The selected observation and interview questions from tested tools were the most critical to measure the quality of care in camps in relation to the non-emergency standard for the minimum package of Primary Health Care services delivered in camp settings. Despite that limitation, it provided a rapid assessment fitting emergency and sensitive contexts in which rapid evidence generation is crucial. Third, some parts of the tools were from a manual written in 2004 and were pretested locally and adjusted afterwards. However, during the analysis, some questions appeared ambiguous on the answers possible, asking for different items within the same question. Potential confounders could not be collected nor tested to limit risks and disturbance on health care provision. This has implication for the validity of the statistical results. Data collectors were trained to ensure the quality of the data, but observation bias, especially for technical competence, were expected and mitigated by having medically qualified enumerators. Other limitations were specific to the setting. The ‘Satisfaction’ domain score was not considered very informative based on its composition, however, it can be improved and better measured in subsequent research. Previous experience and knowledge of current IDPs about health care, prior to the emergency, is unknown and, therefore, their expectation cannot be explored, nor their satisfaction level fully understood. Finally, although it is meant to be a rapid assessment of the quality of care taking place in a difficult context, the representation of the beneficiaries cannot be verified with six respondents per health facility. Correcting for such limitations was attempted using observations and a checklist to assess perceived and realised quality of care.

## Conclusion

This analysis of the quality of care provided in PHCC in IDP camps in Iraq in 2018 in a humanitarian emergency setting shed some light on both the quality of care in camps, and on the method of such assessment. This research attempted to demonstrate that it is possible to measure the quality of care beyond SPHERE standards. It is an important step toward ensuring that IDPs’ dignity is respected by providing them with adequate and quality care. Further research is necessary on both the way to provide such quality of care, as well as to assess it in an emergency context. Nonetheless, such research remains very relevant as most emergencies are long, protracted crises where morbidity reduction rather than mortality reduction becomes the focus of care provision. An essential aspect of the overall quality of care provided in PHCCs is the involvement of the Iraqi government through the DoH. This was possible as only parts of the country suffered from a heavy disruption of services, while the integrity of state authority was maintained. This point is important as many emergencies are localized and governmental function and integrity remain, at least partially, in most contexts. In Iraq, the cooperation between the DoH, international NGO’s, and local NGOs allowed the delivery of primary health care to vulnerable populations, and the possibility to improve the quality of care delivered to IDP camps.

A few recommendations can be extracted from this assessment. Firstly, regular quality-of-care assessments are necessary and need to be thought through, planned, and carried out from the onset of the emergency response by the different cluster leads. Data from these assessments need to be shared immediately with the stakeholders involved to ensure improvement processes take place. Secondly, the cooperation of all implementing partners through the Health Cluster allowed for an overall improvement in the quality of care delivered, and this reinforces the necessity to include all stakeholders in emergency responses [56, 62–64]. Thirdly, this study can help humanitarian organisation, implementing partners, and governments to improve the quality of primary health care delivery in camp settings, both in emergency and post-emergency contexts. Finally, it shows that it is possible to improve the quality of care for beneficiaries beyond the minimum SPHERE standards.

This study provides an important step towards improving the quality of care in camps in Iraq and elsewhere by showing it is possible to assess the quality of care through standardized, non-emergency quality care criteria in emergency settings and can help set up such assessments in similar settings with the hope of providing better and ever-improving health services to displaced people.

### Supplementary Information

**Additional file 1**. Data collection tools: Facility observation, Clinical Observation, Health worker interview, Patient exit interview.

## Figures and Tables

**Fig. 5:**
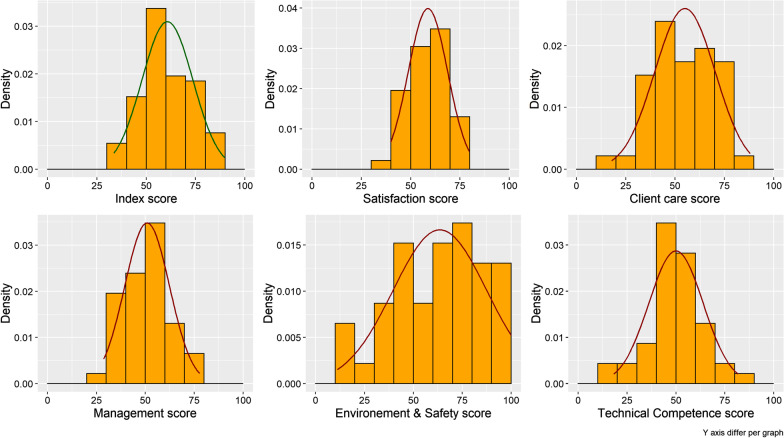
Distribution of index and domains scores, all camps, both phases

## Data Availability

Data were collected by WHO for the health cluster for the camps in Iraq, which was later presented to Iraqi Ministry of Health, partners, and donors for monitoring. Data is available upon reasonable request at the WHO Country Office Iraq.

## Appendix

### Appendix 1 Fig. 5

See Fig. 5.

### Appendix 2: Definitions

#### IDP and camps

The definition of Internally Displaced Person is the following: Persons or groups of persons who have been forced or obliged to flee or to leave their homes or places of habitual residence, in particular as a result of or in order to avoid the effects of armed conflict, situations of generalized violence, violations of human rights or natural or human-made disasters, and who have not crossed an internationally recognized state border. Camps are usually understood as temporary and last resort solutions that offer protection and assistance for population who have been displaced from their homes because of violence, nature- or human-made disasters [44, 65].

#### Health cluster partners

The Health Cluster partners are the supporting organisations and comprise local NGO (NGOs), international NGOs (INGO), and the Directorate of Health, subdivision of the Ministry of Health at the Governorate level.

#### WHO quality of care

- Effective: delivering health care that is adherent to an evidence base and results in improved health outcomes for individuals and communities, based on need.
- Efficient: delivering health care in a manner which maximizes resource use and avoids waste; accessible, delivering health care that is timely, geographically reasonable, and provided in a setting where skills and resources are appropriate to medical need.
- Acceptable/patient-centered: delivering health care which takes into account the preferences and aspirations of individual service users and the cultures of their communities
- Equitable, delivering health care which does not vary in quality because of personal characteristics such as gender, race, ethnicity, geographical location, or socioeconomic status.
- Safe, delivering health care which minimizes risks and harm to service users.

## Abbreviations

CC: Client care
CCCM: Camp coordination and camp management
DoH: Directorate of health
ES: Environment and safety
IASC: Inter-agency standing committee
IDP: Internally displaced persons
I/NGO: International/Non-governmental Organisation
IOM: International office for migration
IRCS: Iraqi Red Crescent Society
MG: Management
PHCC: Primary health care centre
ST: Satisfaction
TC: Technical competence
UNHCR: United Nations high commissioner for refugees
WASH: Water, sanitation and hygiene

## Footnotes

1 An international coordination architecture set at the global level to coordinate humanitarian emergency response, by the IASC and partners.
