# Supplementary material for: An assessment of the quality of care provided at primary health care centres in camps for internally displaced persons in Iraq in 2018
Source: Confl Health. 2021 Sep 8;15:67. doi: 10.1186/s13031-021-00402-4 (PMC8425107; doi:10.1186/s13031-021-00402-4)
Supplement: Supplementary file 1 — Additional file 1. Data collection tools: Facility observation, Clinical Observation, Health worker interview, Patient exit interview. [file 13031_2021_402_MOESM1_ESM.docx]

**ADDITIONAL FILES Quality of Care Iraq**

**Data collection tools**

- 1. Observation checklist

Health facilities observations; physician, nurse, pharmacist, laboratory technician

| **Facility Observation** | | | | | |
| --- | --- | --- | --- | --- | --- |
| **Domain** | **Q No.** | **Question** | **Answers** |  |  |
| Environment and Safety | 1 | There are clearly visible outside signs directing to and marking the Health Facility | **0=No** |  | **2=Yes** |
| Environment and Safety | 2 | The facility has services and working days & hours clearly posted | 0=No |  | 2=Yes |
| Environment and Safety | 3 | There are clear signs inside the center indicating how and where to get services | 0=No |  | 2=Yes |
| Client Care | 4 | There are health education posters distributed at the walls in the waiting area, in the examination rooms and in MCH | 0=none | 1=Partial | 2=all |
| Client Care | 5 | There is a private family planning counseling area | 0=No |  | 2=Yes |
| Client Care | 6 | There is a private oral rehydration corner | 0=No |  | 2=Yes |
| Environment and Safety | 7 | The waiting room has enough functional seats for clients | 0=No | 1=Partial | 2=Yes |
| Environment and Safety | 8 | The facility is clean (equipment, instruments, walls, furniture, floors, toilets, sinks) | 0=No | 1=Partial | 2=Yes |
| Environment and Safety | 9 | All wires and installation outlets are properly covered. | 0=No |  | 2=Yes |
| Environment and Safety | 10 | There is a functioning refrigerators at the Laboratory, Pharmacy and Immunization units | 0=none | 1=Partial | 2=all |
| Environment and Safety | 11 | There is drinking water available for all clients | 0=No |  | 2=Yes |
| Environment and Safety | 12 | There is a functioning water faucets and toilets for clients | 0=No |  | 2=Yes |
| Environment and Safety | 13 | There is a trash can for medical waste and special box for needels at examination rooms | 0=none | 1=Partial | 2=all |
| Environment and Safety | 14 | Disposable gloves are available in each examination room | 0=none | 1=Partial | 2=all |
| Environment and Safety | 15 | Soap and water or cotton swabs with alcohol are available in each examination room | 0=none | 1=Partial | 2=all |
| Environment and Safety | 16 | There is equipment for sterilization | 0=No |  | 2=Yes |
| Management | 17 | Infection Prevention procedures are posted at the emergency and consultation rooms | 0=No |  | 2=Yes |
| Management | 18 | Supply shelves in the pharmacy & MCH are organized by date of expiration with those drugs in front expiring first | 0=No |  | 2=Yes |
| Management | 19 | Pharmaceutical supplies & Contraceptives supplies are kept proprly | 0=No |  | 2=Yes |
|  |  |  |  |  |  |
|  |  |  |  |  |  |
| **Clinical Observation** | | | | | |
|  | | | | | |
| **Physician Observation** | | | | | |
| **Domain** | **Q No.** | **Question** | **Answers** |  |  |
| Technical Competence | 1 | Did the physician follow the Infection Prevention guidelines washing hands before or after the examination? | 0=No |  | 2=Yes |
| Client Care | 2 | Did the physician greet the patient respectfully,Referring to him/her by name Asking to sit down and Looking at patient when talking with him/her | 0=No | 1=Partial | 2=Yes |
| Technical Competence | 3 | Did the physician ask about the client's health and symptoms and medical history? | 0=No |  | 2=Yes |
| Technical Competence | 4 | Did the physician follow the guidelines to do a physical assessment, Weight and height, Oedema check, Blood pressure and Heart and lung sounds | 0=none | 1=Partial | 2=all |
| Technical Competence | 5 | Did the physician examine the client according to his condition? | 0=No |  | 2=Yes |
| Client Care | 6 | Did the physician explain the treatment he was offering and ask the client if they understood and if he has any questions? | 0=No | 1=Partial | 2=Yes |
| Client Care | 7 | Did the physician deliver any of the following health messages and ensure client understood? Diet, smoking, medication, exercise and follow up care | 0=none | 1=Partial | 2=all |
| Client Care | **8** | Did the physician tell the client when to return for the next appointment? | 0=No |  | 2=Yes |
|  |  |  |  |  |  |
| **Nurse Observation** | | | | | |
| **Domain** | **Q No.** | **Question** | **Answers** |  |  |
| Technical Competence | 1 | Did the Nurse follow the Infection Prevention guidelines washing hands before or after the procedure? | 0=No |  | 2=Yes |
| Client Care | 2 | Did the nurse prepare for taking vital signs by :Greeting the patient Informing about the procedure, asking to sit down, using patient's name and Looking at him when speaking and insuring the patient is comfortable | 0=none | 1=Partial | 2=all |
| Client Care | 3 | Did the nurse arrange for patient's privacy? | 0=No |  | 2=Yes |
| **Pharmacist Observation** | |  |  |  |  |
| Client Care | 1 | Did the pharmacist greet the client courteously? | 0=No |  | 2=Yes |
| Technical Competence | 2 | Before dispensing medication, did the pharmacist ask whether the client had any allergies or was on other medication? | 0=No |  | 2=Yes |
| Client Care | 3 | Did the pharmacist give instructions about how to take the drug and confirm client understood the instructions? | 0=No |  | 2=Yes |
| Client Care | 4 | Did the pharmacist describe the potential side effects and how to manage them? | 0=No |  | 2=Yes |
| Technical Competence | 5 | Did the pharmacist write on the package: Patients name, Medicine Name, Dosage and way of administration | 0=none | 1=Partial | 2=all |
|  |  |  |  |  |  |
| **Laboratory Technician Observation** | | | | | |
| **Domain** | **Q No.** | **Question** | **Answers** |  |  |
| Client Care | 1 | Did the lab technician start by greeting the client and explaining the procedure? | 0=No |  | 2=Yes |
| Technical Competence | 2 | Did the provider follow the Infection Prevention guidelines washing hands before putting on gloves? | 0=No |  | 2=Yes |
| Technical Competence | 3 | Did the technician put on gloves before handling specimens? | 0=No |  | 2=Yes |
| Technical Competence | 4 | Did the technician dispose of the used needle and syringe in a sharps container? | 0=No |  | 2=Yes |
| Technical Competence | 5 | Did the technician soak resuable laboratory equipment in a decontamination solution before cleaning? | 0=No |  | 2=Yes |
| Client Care | 6 | Did the technician tell the client when to return for the results? | 0=No |  | 2=Yes |

- 1. Interviews questionnaires

Interviews with health workers and patients

|  | | **Health Worker Interview** | |  |  |
| --- | --- | --- | --- | --- | --- |
| **Q: Do you agree to participate in the survey ?** | | | | **Yes** | **No** |
| **Domain** | **Q No.** | **Question** | **Answers** |  |  |
| Management | **1** | Have you received training in the last year in any of the following. Subjects: |  |  |  |
|  |  | Disease Management | 0=none | 1=1-2 items | 2= > 2 items |
|  |  | Communication skills | 0=none | 1=1-2 items | 2= > 2 items |
|  |  | Work planning | 0=none | 1=1-2 items | 2= > 2 items |
|  |  | Standards of care | 0=none | 1=1-2 items | 2= > 2 items |
|  |  | supervision | 0=none | 1=1-2 items | 2= > 2 items |
|  |  | problem solving | 0=none | 1=1-2 items | 2= > 2 items |
|  |  | reproductive health | 0=none | 1=1-2 items | 2= > 2 items |
|  |  | infection prevention | 0=none | 1=1-2 items | 2= > 2 items |
|  |  | Health Education | 0=none | 1=1-2 items | 2= > 2 items |
|  |  | other: | 0=none | 1=1-2 items | 2= > 2 items |
| Management | 2 | Did the supervisor give you any performance related feedback? | 0=No |  | 2=Yes |
| Management | 3 | Show me the protocols, standards or guidelines you use to guide service delivery in your centre? | 0=not available |  | 2=available |
| Management | 4 | Have you received training in these standards, protocols or guidelines? | 0=No |  | 2=Yes |
| Satisfaction | **5** | How would you rate team work at this centre? | 0=poor | 1=adequate | 2=excellent |
|  |  |  |  |  |  |
|  | | **Patient Exit Interview** | |  |  |
|  |  |  |  |  |  |
| **Q: Are you agree to participate in the survey ?** | | | | **Yes** | **No** |
| **Domain** | **Q No.** | **Question** | **Answers** |  |  |
| Satisfaction | 1 | What was the reason for your visit today? General Physician(1), MCH(2) Follow up(3), others(4) |  |  |  |
| Satisfaction | 2 | Do you find services are offered when you need them? | 0=No | 1=Partial | 2=Yes |
| Satisfaction | 5 | Was there a place for you to sit in the waiting room today? | 0=No |  | 2=Yes |
| Satisfaction | 11 | Have you had a laboratory test at a centre? | 0=No |  | 2=Yes |
| Satisfaction | 12 | If Yes, did the provider explain the results of your test? | 0=No |  | 2=Yes |
| Management | 13 | Have you ever been referred to another Health Facility care? | 0=No |  | 2=Yes |
| Management | 14 | If yes, did the provider give you a note to take to the referral site and to return to the health centre? | 0=No |  | 2=Yes |
| Satisfaction | 17 | Were there people present during your examination who could see or hear you other than your provider? | 0=yes |  | 2=no |
| Satisfaction | 18 | Were you satisfied with the healthcare services you received today? | 0=No |  | 2=Yes |
| Management | **21** | Was the medication prescribed today available? | 0=none | 1=some | 2=all |
| Client Care | **22** | Did the provider make a follow up appointment? | 0=No |  | 2=Yes |
